# Molecular Regulatory Mechanism of Inflorescence, Flower and Fruit Development in Tomato

**DOI:** 10.3390/plants15071064

**Published:** 2026-03-31

**Authors:** Shengjie Ma, Yishan Fu, Xinlei Du, Jie Zhang, Qing Gao, Junfeng Zhang, Xingren Shi, Aoxue Wang, Lei Cao

**Affiliations:** 1 College of Horticulture and Landscape Architecture, Northeast Agricultural University, No. 600, Changjiang Road, Xiangfang District, Harbin 150030, China; 2 School of Geography and Tourism, Harbin University, No. 109, Zhongxing Avenue, Nangang District, Harbin 150076, China; 3 Wuxing Jinnong Ecological Agriculture Development Co., Ltd., Huzhou 313000, China

**Keywords:** tomato, floral organs, MADS-box genes, reproductive development, ABCDE model, flower–fruit continuum, molecular design breeding

## Abstract

Tomato (*Solanum lycopersicum* L.) is a globally important vegetable crop and a key model species for studying reproductive development in other Solanaceae members with edible fleshy fruits, such as eggplant, sweet and hot peppers, and *Physalis* spp. The morphogenesis and patterning of tomato floral organs fundamentally determine fruit yield and quality. Recent advances in high-throughput sequencing and gene editing have significantly deepened our understanding of the molecular network regulating tomato reproductive development. This process, from the transition of vegetative shoot apical meristem to the inflorescence meristem, forming floral meristems with primordia of sepals, petals, stamens, carpels, and fruits, is precisely coordinated by a genetic network involving homeobox and other types of transcription factors, along with signaling pathways. This review systematically outlines the core regulatory network, with an emphasis on the MADS-domain transcription factor family and its associated ABCDE model. Integrating insights from hormone signaling and mutant phenotypes, we summarize the maintenance of inflorescence meristem identity, the specification of floral meristems, and the morphogenetic patterns and core gene regulatory mechanisms for each floral whorl in tomato. We further extend this framework to the flower–fruit continuum, examining how carpel development, floral meristem termination, and ovule differentiation influence fruit morphology, locule number, pericarp structure, and metabolic traits. Finally, we discuss the integration of floral organ development with molecular design breeding and formulate a forward-looking research agenda that translates floral regulatory mechanisms to breeding strategies for yield, uniformity, and fruit quality. This synthesis provides a theoretical foundation and genetic resources for the genetic improvement of tomato flower architecture and its underlying regulatory mechanisms.

## 1. Introduction

Tomato (*Solanum lycopersicum*), a leading global vegetable crop, is predominantly enhanced through hybrid breeding to develop fruits with superior resistance, yield, and quality [1]. Floral organs serve as critical determinants of fruit set, quality, and yield potential, with their morphology, fertility and pollination efficiency of the corresponding floral organs play a decisive role in determining fruit set and the qualitative and quantitative characteristics of fruits. As typical actinomorphic flowers, tomato flowers exhibit a radial symmetry that facilitates efficient pollen transfer. In addition, male-sterile lines are indispensable for hybrid seed production in this species. Distinct floral phenotypes (such as exserted stigma and multi-petal flowers) offer valuable models for investigating developmental regulation. Hence, a systematic understanding of the molecular networks governing floral patterning, fertility determination, and pollination regulation in tomato offers a fundamental theoretical framework to guide its genetic improvement.

Floral development in tomato is a complex morphogenetic process under genetic regulation. It initiates with the transition of the shoot apical meristem to an inflorescence meristem, which subsequently generates floral meristems. The cells of the floral meristem (FM) differentiate to form four canonical whorls of floral organs from periphery to center: sepals, petals, stamens, and carpels [2,3]. The precise regulation of this process not only affects floral fertility but also directly determines fruit-related traits, such as size, locule number, morphology, and ripening characteristics, through its influence on ovary and ovule development [4]. Therefore, elucidating the molecular mechanisms underlying tomato flower development is essential for targeted improvement of fruit traits. Accumulating evidence indicates that transcription factor-mediated gene regulatory networks form the core framework for floral organ development, wherein the MADS-box family functions as a key regulatory module [5].

The MADS-box transcription factor family is a key regulator of floral organ identity, operating primarily through the ABCDE model established in *Arabidopsis thaliana*. Building on this framework, the model Quartet further proposes that MADS-domain proteins form heterotetrameric complexes, such as AAEE for sepals, AB1B2E for petals, B1B2CE for stamens, CCEE for carpels, and CDE combinations for ovules, to regulate downstream target genes and specify floral organ identity. These MADS-domain tetramers recognize CArG-box elements (CC(A/T)_6_GG) in the promoters of target genes [6]. In this model, B1 and B2 denote the AP3- and PI-lineage proteins, respectively, which are both required for B-class function. Homologs of these Arabidopsis ABCDE genes have been well characterized in tomato and many other plant species. In tomato, the corresponding ABCDE genes have been identified: Class A includes *MACROCALYX* (*MC*) and *APETALA2* (*AP2*) (AP2/ERF family transcription factor) [7]; Class B genes in tomato comprise two *AP3*-like genes, *TOMATO APETALA3* (*TAP3*) and *TOMATO MADS-BOX 6* (*TM6*), representing the B1 (*AP3*) class, and two *PI*-like genes, *Lycopersicum esculentum PISTILLATA* (*LePI*; also designated *SlGLO1*) and *TOMATO PISTILLATA* (*TPI*; also designated *SlGLO2*), representing the B2 (*PI*) class [8,9]. In the Quartet model, B1 and B2 proteins form obligate heterodimers that assemble into higher-order complexes to specify petal and stamen identity [8]; Class C is represented by *TOMATO AGAMOUS 1* (*TAG1*) and *TOMATO AGAMOUS-LIKE 1* (*TAGL1*), the two characterized C-type MADS-box genes in tomato [10]; Class D consists of *Sl-AGL11* and *Sl-MBP3* (two homologs of Arabidopsis *SEEDSTICK* (*STK*)) [11]. These genes may function in ovule development together with another tomato gene, *SISTER OF TOMATO MADS-BOX 3* (*STM3*, ortholog of *ARABIDOPSIS BSISTER* (*ABS*)) [12]. In *A. thaliana*, ABS and STK have been shown to play a fundamental role, even before fertilization, in the molecular interactions between the integument and the female gametophyte [13]. Class E includes *TOMATO MADS-BOX 29* (*TM29*), *TOMATO MADS-BOX* (*TM5*), *S. lycopersicum MADS-BOX PROTEIN 21* (*SlMBP21*), *JOINTLESS 2* (*J2*), and other *SEPALLATA*-like genes [5,14,15].

Key flowering-time genes in tomato are largely orthologs of Arabidopsis regulators. For instance, *SINGLE FLOWER TRUSS* (*SFT*), the ortholog of *FLOWERING LOCUS T* (*FT*), promotes flowering under both long- and short-day conditions, and its function is partially redundant with *FALSIFLORA* (*FA*), the ortholog of *LEAFY* (*LFY*) [15,16]. Other critical regulators include *SELF PRUNING* (*SP*) (ortholog of *TERMINAL FLOWER1* (*TFL1*), which controls inflorescence determinacy [16]; *MACROCALYX* (*MC*, or *LeMADS*-*MC*), homolog of *APETALA1* (*AP1*), involved in sepal development and the flowering transition [17]; and the orthologs of *SUPPRESSOR OF OVEREXPRESSION OF CONSTANS1* (*SOC1*), *SHORT VEGETATIVE PHASE* (*SVP*), and *JOINTLESS* (*J*) [5]. It is noteworthy that the development of the four floral whorls is not solely governed by the MADS-box transcription factors, but also involves other functional genes. For example, among the genes mentioned in this paragraph, *LFY*/*FA*, *FT*/SFT and *TFL1*/*SP* are not members of the MADS-box family. Another example is *SlGT11*, which encodes a transcription factor of the Trihelix family and controls floral organ patterning and the determinacy of floral organ arrangement [18].

Tomato flowering culminates in the formation of a fleshy fruit, which includes all stages of gynoecium development, from carpel/ovule initiation to fruit ripening, including under the control of MADS-box genes of the C (*TAG1* and *TAGL1*), D (*Sl-AGL11* and *Sl-MBP3*) and E (e.g., *TM29* (*TAGL2*), *TM5* (*TDR5*), *RIPENING INHIBITOR* (*RIN*)) classes [14,19,20,21]. Simultaneously, plant hormones, such as auxins and gibberellin (GA), can interact extensively with these transcriptional networks via their components (ARFs, DELLAs), forming spatiotemporally specific regulatory modules [22].

The endpoint of tomato floral organ development is not the flower itself, but rather the initiation and completion of fruit formation. The cell fate of carpel primordia established during floral development, the differentiation status of ovules, the timing of floral meristem inactivation, and inflorescence structural characteristics collectively constitute the developmental framework that predetermines fruit morphology, size, texture, and metabolic quality [23,24]. In recent years, with deepening insights into internal replication control, cell cycle exit decisions, and the spatial distribution of ploidy levels, the molecular basis for fruit wall cell expansion and locule number determination has been progressively elucidated to the level of direct interactions between transcription factors and cell cycle machinery [24,25]. These advances suggest that key targets for fruit trait improvement exist not only during fruit development but should be traced back to cell fate decisions during floral organogenesis.

For this, we propose the following hypothesis: tomato floral development is functionally continuous with fruit trait specification—fruit size, structure, and quality represent delayed outputs of floral patterning decisions. To evaluate this hypothesis based on existing evidence, we address three objectives: (1) establish the core MADS-domain transcriptional network establishing floral organ identity; (2) extend this framework to the flower–fruit transition, linking carpel development to fruit morphology and pericarp structure; (3) integrate hormonal and environmental signals modulating this developmental continuum.

This review summarizes current advances in the study of molecular regulation of tomato floral development, spanning inflorescence architecture to the specification of floral organ identity. We focus on the core mechanisms governing the development of each floral organ, like sepals, petals, stamens, pistils, and stigma, with an emphasis on protein interactions and associated signaling pathways. By integrating these insights, this article aims to establish a clearer framework for understanding floral organ development and to inform future strategies for the targeted improvement of flower morphology in tomato.

## 2. Flower Structure in Tomato

Tomato flowers are arranged in a cyme inflorescence, with each inflorescence typically bearing 5–8 flowers [26]. Each individual flower is a perfect, actinomorphic (radially symmetrical), composed of four whorls of organs. The outermost whorl consists of five green sepals, which serve a protective function. The next inner whorl comprises five yellow petals that attract pollinators. The third whorl contains five stamens, whose anthers are laterally fused to form a distinctive tubular structure (anther cone), a key feature enabling cleistogamy (closed pollination) in tomato. The pistil is composed of a minimum two carpels, and each carpel is composed of the ovary, style, and stigma. The number of carpels determines (is equal to) the number of locules in the upcoming tomato fruit. The stigma is typically recessed within the anther cone, ensuring highly efficient self-pollination [27].

The following sections summarize recent research advances within this developmental framework.

## 3. Mechanisms Regulating the Onset of Flowering, Inflorescence Architecture and Floral Organ Development

### 3.1. Regulation of Inflorescence Architecture

The transition of the shoot apical meristem from the vegetative to the reproductive state is under the control of several key transcription factors, a large number of which belong to the MADS-domain family. These transcription factors regulate not only the flowering time but also the inflorescence architecture (a key factor of plant productivity) via the identity, localization and number of the differentiating floral meristems. The tomato MADS-box gene *SFT*, encoding the ortholog of the Arabidopsis florigen *FT*, plays a key stimulating role in flowering initiation. Another tomato MADS-box gene *SP*, encoding an ortholog of the Arabidopsis *TFL*1, negatively controls the transition to the reproductive period and regulates the ‘determinate’ (*sp*/*sp*) and ‘indeterminate’ (*SP*−) inflorescence type [28]. Specifically, *SFT* is a central promoter of flowering initiation and influences floral meristem identity, number, and size [16,29]. These two MADS-box genes act in a parallel pathway with *FA*, the ortholog of *LFY*, and both the *SP*/*SFT* and *FA* pathways are essential for successful flowering in tomato [16,28,29]. The study found that MADS-domain protein MC (AP1 ortholog), interacts with SFT and MADS-domain transcription factor J (SVP ortholog), to coordinate inflorescence meristem fate and architecture [17]. STM3 and J2 are tomato homologs of Arabidopsis SOC1 and SEPALLATA4 (SEP4), respectively, and have opposing regulatory functions in controlling inflorescence branching [12]. STM3 complexes with J2 to control branching by activating MADS-box gene *FRUITFULL1* (*FUL1*); mutations in these genes result in aberrant inflorescence structures [12,30]. Other *FRUITFULL*-like genes, such as *FUL2* and *MBP20*, suppress excessive branching by promoting the maturation of floral meristems [31]. Beyond the MADS-box network, the miR156-SPL module, particularly miR156a targeting *SlSPL13*, modulates flowering time and inflorescence structure partly through direct regulation of *SFT* [32]. Furthermore, the TCP family transcription factor SlTCP26 promotes lateral branch development, contributing to the overall plant architecture [33]. Collectively, these components constitute an integrated regulatory network, ensuring the proper maintenance and developmental transition of the inflorescence meristem [34,35].

### 3.2. Floral Meristem Identity Determination and Primordium Initiation

The formation of the FM is fundamental for subsequent floral organogenesis and is regulated by many key factors other than MADS transcription factors. WUSCHEL (WUS) is a homeodomain transcription factor necessary for the maintenance of the stem cell niche in the shoot apical, inflorescence and floral meristems. WUS is specifically expressed in the meristem organizing center and, through the CLAVATA-WUSCHEL (CLV-WUS) pathway, controls proper meristem and floral organ development. At the end of flower development with the formation of carpels, ovaries and ovules, the expression of *WUS* is suppressed by the combined action of C-class MADS-box transcription factor AGAMOUS with cofactors (including MADS-box proteins of D and E classes). In tomato, the ortholog of WUS is SlWUS, which is expressed in the FM and required for meristem maintenance and proper floral organ development; complete loss-of-function of SlWUS leads to a decrease in the number of carpels and, thus, fruit locules [36,37]. In contrast, INHIBITOR OF MERISTEM ACTIVITY (IMA) functions as a repressor of *WUS* to fine-tune meristematic activity during flower and ovule development [38]. Auxin signaling is pivotal for initiating floral primordia. Transcription factors such as SlARF5 participate in this process while concurrently regulating plant stature [39]. Studies show that auxin, via its response factors (ARFs), generates local concentration maxima that precisely determine the sites of primordium emergence [40]. Furthermore, the LITTLE ZIPPER protein DTM regulates shoot apical meristem function by demarcating expression boundaries of key meristem genes [41]. The AP2/ERF family transcription factor, ENO, modulates floral meristem size and organ number through the CLV-WUS pathway, representing an important evolutionary node for fruit size variation [42]. Epigenetic regulation also contributes: the histone acetyltransferase SlGCN5, for instance, sustains meristem activity and influences floral development by regulating the expression of central regulators like WUSCHEL [43].

### 3.3. Regulation of Sepal Development

**Figure 1:**
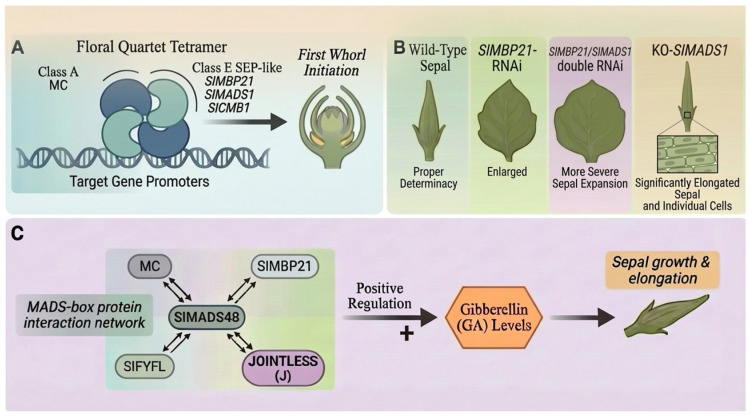
Regulatory network controlling tomato sepal development. (**A**) Core combinatorial MADS-box complex: Class A (MC) and class E proteins assemble into a floral quartet tetramer to specify sepal identity and floral determinacy. (**B**) Morphological comparison between wild-type and mutant. (**C**) Modulation of sepal growth: SlMADS48 interacts with MC, SlMBP21, SlFYFL, and J to promote sepal elongation by elevating GA levels. (Arrows indicate positive regulatory effects or developmental promotion, Double-headed arrows indicate protein–protein interactions). Image was created with BioGDP (https://BioGDP.com (accessed on 8 January 2026)) and enhanced by DeepSider (https://web.deepsider.app/chat/ (accessed on 23 March 2026)).

Differentiation of the floral meristem begins from the periphery, where the initiation of sepals occurs. Sepals constitute the first floral whorl and are specified by class A genes acting in concert with class E genes (Figure 1A). In tomato, the *MC* is required for proper sepal development and inflorescence architecture. The tomato ripening-inhibitor (*rin*) mutant shows enlarged sepals, which are controlled by *LeMADS*-*MC*, one of the two tandem MADS-box genes at the rin locus whose expression is altered by the *rin* mutation [44]. Class E *SEP* genes, including *SlMBP21*, *SlMADS1*, and *SlCMB1*, cooperate in sepal development; mutations or silencing of these genes lead to severe sepal malformations or abnormal morphology [45,46,47] (Figure 1B). Beyond class A genes, other MADS-box genes are also involved in sepal development. For example, SlMADS48 has been shown to interact with several known MADS-box proteins involved in sepal development (MC, SlMBP21, SlFYFL, and J). Overexpression of *SlMADS48* results in elongated sepals and elevated GA levels in tomato, indicating its positive role in regulating sepal growth [48] (Figure 1C).

### 3.4. Regulation of Petal Development

**Figure 2:**
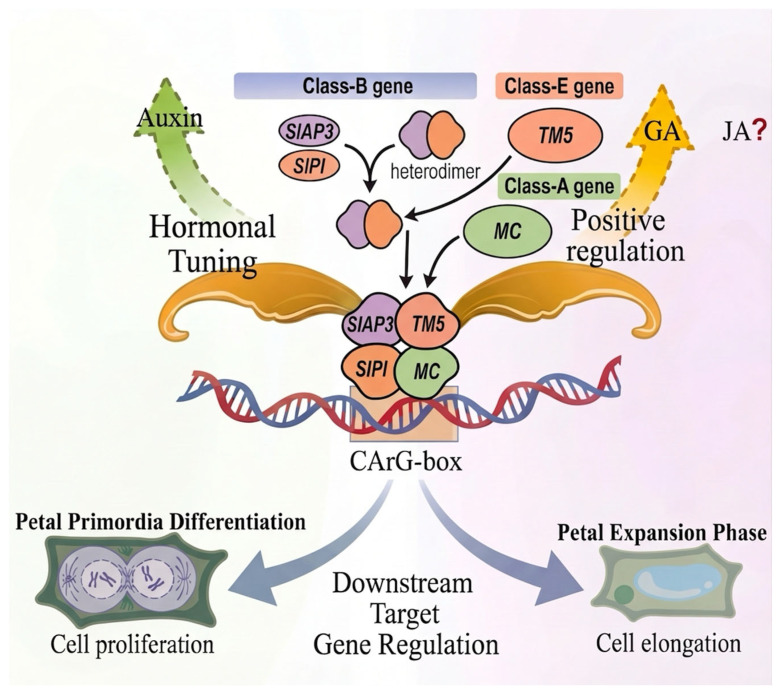
Core regulatory network controlling tomato petal development. Class B MADS-box proteins (SlAP3 and SlPI) form a heterodimer that assembles with the E-class and the class A protein MC into floral quartet tetramers (Black arrows indicate assembly of the floral quartet tetramer). These tetramers bind to CArG-box cis-regulatory elements in target gene promoters to specify petal identity. Auxin and GA may positively regulate petals, while JA is associated with petal regulation (dashed arrows indicate inferred relationships, whereas solid arrows represent confirmed regulation). This spatiotemporally regulated network governs final petal size and morphology. Image was created with BioGDP (https://BioGDP.com (accessed on 8 January 2026)) and enhanced by DeepSider (https://web.deepsider.app/chat/ (accessed on 23 March 2026)).

Unlike Arabidopsis, where most floral organ identity genes are present as single copies, tomato exhibits extensive gene duplication with subsequent subfunctionalization. The B-class MADS-box genes exemplify this pattern: while Arabidopsis possesses single *AP3* and *PI* genes, tomato harbors two AP3 paralogs (*TAP3* and *TM6*) and two PI paralogs (*SlGLO1* and *SlGLO2*). Functional analyses reveal that *TM6* has acquired a broader expression domain extending into carpels—a feature not observed in *Arabidopsis AP3*—indicating expression domain diversification following duplication [8,9,49]. As the second whorl of floral organs, the primary function of petals is to attract pollinators through their specific size, color, and morphology while also providing physical protection for the stamens and gynoecium. The development of tomato petals is controlled by a core regulatory network dominated by class B MADS-box genes, which are divided into the *SlAP3* subfamily (*TAP3*, *TM6*) and the *SlPI* subfamily (*SlGLO1, SlGLO2*) [5,49]. The SlAP3 and SlPI proteins form a heterodimer that can interact with both the tomato SEP3 ortholog (TM5, or SlMADS5) and the tomato class A protein MC, presumably forming tetramer to specify petal identity [50] (Figure 2). *TM6* acts partially redundantly with *TAP3* while also exerting unique functions. The EMS-derived loss-of-function mutant succulent stamens 2 (*sus2-1*) carries a mutation in *TM6* and exhibits clear defects in petal development and homeotic transformation of stamens towards carpel-like identity [9]. The *TAP3* loss-of-function mutant (*tap3*) shows a complete transformation of petals into sepal-like structures (green and leathery) and stamens into carpel-like structures [49]. The final size and morphology of petals are fine-tuned by multiple hormones, petal development is affected by gibberellins, auxin, and jasmonic acid [51].

### 3.5. Regulation of Stamen Development

As discussed above, stamen identity is established through the synergistic action of MADS-domain transcription factors of classes B, C, and E. The resulting tetrameric B1B2CE complexes activate key downstream regulators. While *SlAP3* and *SlPI* expression is largely confined to petals and stamens, *TM6* shows a broader expression pattern that includes carpels, reflecting functional divergence [9,52]. Mutant analyses highlight their roles: *TAP3* mutants develop carpelloid stamens [49], the *carpelloid stamen and parthenocarpy* (*csp*) mutant, exhibits significant downregulation of *TAP3* expression in petals and stamens [53], while mutations in *SlGLO2* or *TM6* lead to carpelloid stamens and male sterility [52,54]. Therefore, a precise balance of class B and C activities, mediated through complexes like *B1B2CE*, is essential for correct stamen identity and function.

**Figure 3:**
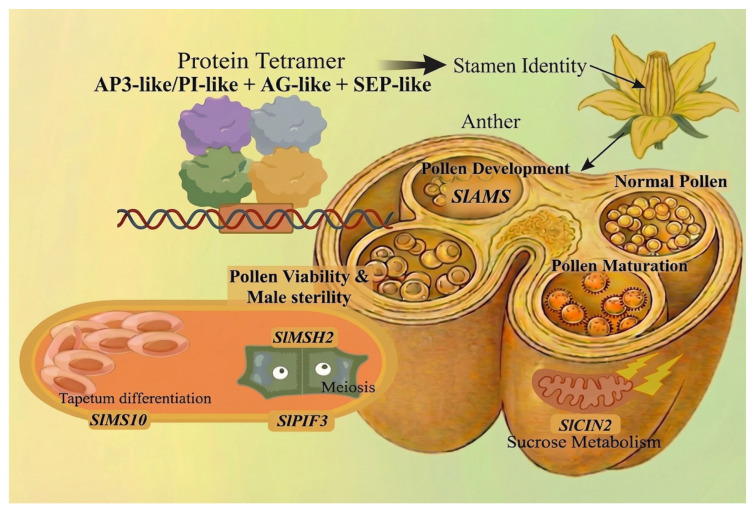
Hierarchical transcriptional and metabolic network controlling stamen development. Stamen identity is established by a MADS-box protein tetramer (AP3-like/PI-like + AG-like + SEP-like), Anther and pollen development are orchestrated by a cascade of specialized genes.Thin arrows represent targeting; thick arrows indicate determination. Image was created with BioGDP (https://BioGDP.com (accessed on 8 January 2026)) and enhanced by DeepSider (https://web.deepsider.app/chat/ (accessed on 23 March 2026)).

Subsequent anther and pollen development involves a cascade of specialized genes (Figure 3). *SlAMS* is essential for pollen development in tomato, as both downregulation and upregulation of this gene severely reduce pollen viability and cause abnormal pollen morphology [55]. Meiotic fidelity relies on factors such as the DNA repair gene *SIMSH2* and the transcription factor *SIPIF3*; their mutations cause meiotic arrest or failure of pollen mitosis I, respectively [56]. Energy metabolism is also crucial, SlCIN2 interacts with the sucrose transporter SlSUT2 to modulate sucrose/hexose balance, thereby affecting tapetal programmed cell death and pollen maturation via Abscisic acid (ABA) and ROS signaling [57].

Proper pollen development requires tightly coordinated tapetal function and meiotic progression. The gene *SlMS10* is critical for early tapetum development, and its disruption leads to tapetal dysfunction and subsequent microspore degeneration [58]. Meiotic integrity is maintained by factors such as the DNA mismatch repair gene *SIMSH2* and *SIPIF3*; mutations in these genes cause meiotic arrest or failure of the first pollen mitosis (PMI), respectively, ultimately blocking the production of viable pollen [56,59]. 

Additionally, jasmonic acid (JA) signaling is required for pollen maturation and vitality, as JA-insensitive *jai1-1* mutants exhibit reduced pollen viability and premature anther dehydration [60]. ABA also contributes to normal pollen development, in which *SlNCED1*—encoding a key rate-limiting enzyme for ABA biosynthesis—shows high expression in anthers and pollen. Both overexpression and silencing of *SlNCED1* disrupt pollen development and germination, confirming the essential role of ABA homeostasis in anther and pollen maturation [61]. ABA is a carotenoid-derived hormone, and carotenoid biosynthetic genes, including *PSY1*, are direct targets of the MADS-box factor *RIN* [62,63].

### 3.6. Regulation of Gynoecium Development

The gynoecium—comprising the ovary, style, and stigma—constitutes the fourth floral whorl and is essential for fertilization and fruit set. Carpel identity is specified by class C and E MADS-box genes [5,10,14,15,20], target genes of which regulate organ architecture by recruiting specific transcription factors.

Wu et al. identified genes of three homeodomain-leucine zipper IV (HD-Zip IV) transcription factors that coordinately promote the formation of interlocking trichomes at the anther margin to unite neighboring anthers, generating a closed anther cone and cleistogamy (flower morphology necessitating strict self-pollination). The three HD-Zip IV transcription factors mentioned above, which are involved in determining anther architecture, also control style length by regulating the transition from cell division to endoreduplication [64]. The expression of these HD-Zip IV genes and their downstream gene, *Style 2.1* [65], was sequentially modified to shape the cleistogamy morphology during tomato evolution and domestication [64].

**Figure 4:**
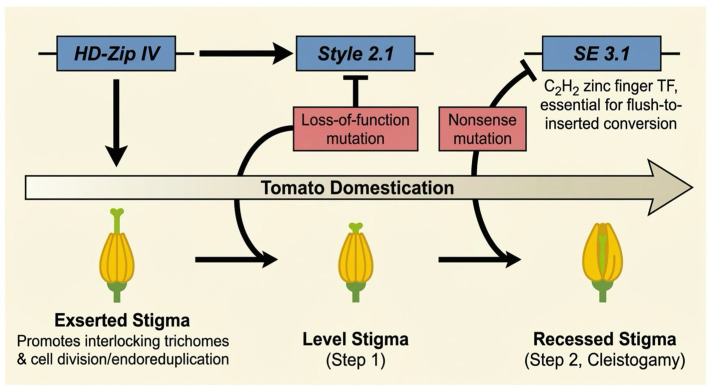
Regulatory cascade controlling stigma position and cleistogamy during tomato domestication. Three HD-Zip IV transcription factors act upstream of *Style 2.1* to coordinate anther cone formation and style length. A loss-of-function mutation in *Style 2.1* drives the transition from exserted to level stigma, followed by a nonsense mutation in *SE 3.1* (encoding a C_2_H_2_-type zinc finger transcription factor) that converts level stigmas to fully recessed stigmas. Straight arrows indicate positive regulation or promotion, whereas the T-bar symbols indicate mutational loss-of-function, leading to a phenotypic transition during tomato domestication. Image was created with BioGDP (https://BioGDP.com (accessed on 8 January 2026)) and enhanced by DeepSider (https://web.deepsider.app/chat/ (accessed on 23 March 2026)).

A key domestication trait in cultivated tomato is the recessed stigma position within the tubular anther cone, which facilitates cleistogamy. Recent studies have revealed a two-tiered genetic cascade governing stigma exsertion [66]. The first transition, from exserted to level stigma, is mediated by a loss-of-function mutation in *style 2.1*. The second transition, from level to fully recessed stigma, involves a nonsense mutation in *SE 3.1*, which encodes a C_2_H_2_-type zinc finger transcription factor essential for the conversion from flush to inserted stigmas [66]. The transition from exserted to recessed stigma is a key innovation enabling efficient self-pollination in cultivated tomato (Figure 4).

Stigma receptivity is a critical indicator of pistil development, which depends on the differentiation of stigma epidermal cells and the production of stigmatic exudate. *SlSTIG1* encodes a small, cysteine-rich protein specifically expressed in the tomato pistil and is abundantly secreted into the stigmatic exudate as a processed 7-kDa peptide. This mature peptide binds to the pollen receptor kinase LePRK2 and to phosphatidylinositol 3-phosphate (PI3P) on the pollen tube surface, thereby promoting in vivo pollen tube elongation. Silencing of *SlSTIG1* significantly reduces the pollen tube growth rate and seed production, confirming its essential role in pollen–pistil interactions and successful fertilization [67].

### 3.7. Regulation of Tomato Ovary Development

In tomato, the ovary is the basal, ovule-bearing part of the gynoecium, which itself constitutes the fourth floral whorl. The tomato gynoecium is typically composed of two carpels, and each carpel’s (including the ovary and ovules) identity is specified by class C, D and E MADS-box genes [5,10,14,15,20]. Carpel number is determined by the size of the FM domain dedicated to pistil formation. This process includes CLV-WUS feedback signaling, which has also been shown in tomato [36,37]. The *S. lycopersicum* gynoecium domain size is tightly regulated, among other factors, by the CRABS CLAW (SlCRC) orthologs (YABBY family transcription factors) [68]. An increased number of carpels as a trait of multilocular fruit in *S. lycopersicum* is associated with two QTLs, *locule number* (*lc*) and *fasciated* (*fas*) [69]. The *lc* locus is located between the *SlWUS* gene and a gene of the WD40 protein, a homolog of the chromatin-remodeling factor 1 subunit, which positively regulates *WUS* expression [70]. Due to the *lc* mutation, the two-locular tomato fruit became four-/six-locular. Against the lc background, the fas mutation led to an even greater number of carpels (>6) [70]. The *FAS* gene encodes a transcription factor of the YABBY2 subfamily, and multilocular fruit is associated with nonfunctional *SlFAS* [71].

**Figure 5:**
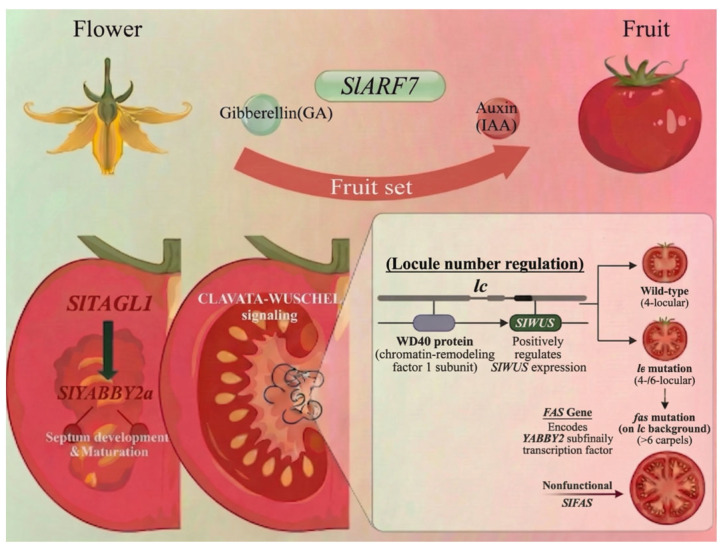
Regulatory network of tomato carpel development and fruit locule formation. Schematic overview of key molecular pathways controlling tomato gynoecium patterning, carpel number, and subsequent fruit set. Hormonal control: GA and auxin signaling via *SlARF7* regulate the transition from flower to fruit set. Septum development and Locule number regulation, please refer to the text. Image was created with BioGDP (https://BioGDP.com (accessed on 8 January 2026)) and enhanced by DeepSider (https://web.deepsider.app/chat/ (accessed on 23 March 2026)).

The functionally partially redundant SlCRCa and SlCRCb are key regulators of floral meristem determinacy. They interact directly with a chromatin remodeling complex (involving SlKNU, SlIMA, SlTPL1, SlHIDA1) to participate in the epigenetic repression of the stem cell gene *SlWUS*, thereby terminating stem cell activity in a timely manner to ensure proper carpel development [68]. Another YABBY member, *SlYABBY2a*, has been identified as a specific regulator of septum development and maturation. Its loss of function leads to septum invagination and delayed maturation, which indicates functional diversification within the YABBY family during carpel formation. The upstream regulator, *SlTAGL1*, regulates the promoter of *SlYABBY2a*, thereby translating floral organ identity signals into concrete morphogenesis [72] (Figure 5).

The *ENO* gene regulates fruit size through the floral meristem development network [42]. Gene editing of the cell cycle transcription factor *SlMYB3R3* can induce longer fruit shape [73], while overexpression of D-class MADS-box gene *SlAGL11* significantly affects flesh tissue differentiation and structure [11]. Recent high-resolution spatiotemporal transcriptome maps have provided detailed evidence for understanding dynamic gene expression during the continuous process from flower development to fruit ripening [74,75]. These studies indicate that many factors acting during floral organ development, such as MADS-box genes (*RIN*, *SlMADS1*), NAC-family transcription factor genes (*SlNAP1*, *NOR*-like1), and *ARFs*, maintain their functions into the fruit development and ripening stages, enabling precise of developmental programs [74,75,76,77]. YABBY family genes are closely associated with fruit shape [78]. *ASYMMETRIC LEAVES 2* (*AS2*) and its homolog *ASL* are crucial for fruit development [79]. NAC transcription factors, like *NOR*-like1, directly regulate fruit size [80]. The DOF transcription factor *SlDOF10* regulates vascular tissue formation during ovary development [81]. In addition, AP2/ERF family transcription factors, such as *SlAP2a*, negatively regulate fruit ripening [82].

In tomato, as in Arabidopsis, the C-function is carried out by two genes of the *AG* subfamily: *TAG1* is an ortholog of *AG* (the euAG lineage) and *TAGL1* is an ortholog of *SHATTERPROOF* (*SHP1*/*SHP2*) (the PLENA (PLE) lineage) [10,83,84]. The phylogenetic separation of the genes corresponds to the separation of the C-function between them: *TAG1* retains the canonical C-class role in specifying stamen and carpel identity, while *TAGL1* has acquired a predominant function in fruit ripening regulation [20,85]. Accordingly, *TAG1* RNAi lines exhibit defects in stamen and pistil development, whereas *TAGL1* silencing causes no floral organ identity defects but severely impairs fruit ripening [12,20,30]. Thus, *TAG1* may influence the fruit structure at the stage of androecium and gynoecium specification, whereas *TAGL1* is more associated with the regulation of fruit characteristics already at the stage of its growth and ripening.

Collectively, the molecular mechanisms governing tomato carpel/pistil development illustrate a distinctive, fruit-centric regulatory network within the Solanaceae. This network embodies an evolutionary adaptation where floral organogenesis is seamlessly and directly coupled with the developmental programs of the subsequent fruit, a feature that stands in contrast to the model plant *Arabidopsis*. This direct linkage underscores why understanding carpel development is fundamental to manipulating fruit architecture.

### 3.8. Inflorescence Architecture Determines Fruit Set and Fruit Size Uniformity

The inflorescence architecture of tomato (i.e., the number of flowers per truss and the degree of branching) directly influences fruit set and the uniformity of mature fruit size, due to competition among developing flowers for limited photosynthetic assimilates.

An increase in the number of flowers per inflorescence may enhance yield potential; however, excessive branching often compromises fruit fertility. For instance, the MADS-box transcription factors *STM3* and *J2* act antagonistically to control inflorescence meristem determinacy and branch number, with *STM3* promoting and *J2* repressing inflorescence branching through direct regulation of *FUL1* [12,30]. These findings suggest that uncontrolled branching directly reduces fruit set.

Furthermore, inflorescence structure determines fruit size uniformity. The *FRUITFULL*-like genes *FUL2* and *MBP20* promote the vegetative-to-reproductive transition and regulate inflorescence architecture [31]; mutations in these genes alter branching patterns, potentially affecting the allocation of photosynthetic products among individual flowers within an inflorescence. In addition, the balance between the florigen gene *SFT* and its antagonist *SP* serves as a master switch governing determinacy versus indeterminacy of meristem activity [86], ultimately determining flower number and the synchrony of flower development.

Therefore, modifying inflorescence architecture represents a key breeding objective to enhance fruit set and achieve the fruit uniformity required for commercial production.

### 3.9. From Flower to Fruit: Endomitosis, Cell Cycle Regulation, and Fruit Size and Peel Structure

The MADS-box transcription factor RIN is a master regulator of tomato fruit ripening, controlling virtually all aspects of this process [62,63,87]. RIN directly activates genes encoding ethylene biosynthesis enzymes (ACS2, ACS4), thereby initiating ethylene signaling that coordinates downstream ripening events [62,87]. Furthermore, RIN directly binds promoters of carotenoid biosynthetic genes (including *PSY1* gene, encoding a chromoplast-specific phytoene synthase), regulating both carotenoid accumulation and the production of ABA—a carotenoid derivative that influences stress responses and developmental processes [62,63]. RIN also regulates sugar metabolism and cell wall modification genes, linking MADS-box activity to multiple facets of fruit quality [63,87].

The final size and shape of tomato fruits are fundamentally determined by the number of carpel primordia cells established during early floral development [88] and subsequent cellular volume expansion during development [89]. Among these, endoreplication—a process of selective nuclear genome amplification without cell division—is the core cytological mechanism driving tomato fruit wall cell enlargement [24,25]. Through peel cell-type-specific transcriptomics, Tourdot et al. analyzed the molecular identity of cells at different ploidy levels. They found that high-ploidy cells significantly enriched genes related to cell wall modification, primary metabolism, and stress response, while low-ploidy cells maintained higher mitotic activity and expressed chromatin modification factors [24]. This study revealed a quantitative correlation between endomitosis levels and pericarp cell identity specialization, demonstrating significant variation among tomato cultivars and dose-dependent regulation by GA signaling [25].

From a floral development perspective, the determination and expansion potential of pericarp cells are partially predetermined during carpel differentiation in the organogenesis phase of flower development. Terao et al. discovered through cell wall polysaccharide immunolocation that the cell expansion pattern during early fruit peel development after tomato pollination is closely linked to the cell wall composition of the floral central carpel primordium. The spatial distribution patterns of pectin and hemicellulose are inherited and amplified during the flower-to-fruit transition [90]. This finding suggests that the genetic network regulating the timing of floral organ cell cycle termination, the threshold for endomitosis initiation, and cell wall remodeling potential serves as a crucial molecular bridge linking floral development to fruit traits. Cell cycle-dependent kinase inhibitors (*KRP*), *CCS52A* activators, and members of the *E2F* transcription factor family have been demonstrated to participate in the regulation of endoreplication initiation [23]. Their spatiotemporal expression patterns in floral organs are directly associated with the final ploidy distribution of pericarp cells and fruit size.

## 4. Synergistic Actions of Hormonal Signaling and Environmental Inputs in Floral and Early Fruit Development

In tomato reproductive development, phytohormones—particularly GA and auxin—function as core signaling modules coordinating floral organ patterning and fruit initiation. Auxin signaling is transduced through Aux/IAA repressors and ARF transcription factors [91,92]. *SlARF7* and *SlARF9* play pivotal roles in fruit set and early fruit expansion [93,94]. Additional transcription factors, including bZIP, DOF, and bHLH members such as *SlPRE2*, further connect GA signaling with floral organ-specific development [95,96].

Importantly, MADS-domain transcription factors directly influence hormone metabolism and signaling, positioning them as central integrators of the hormonal networks that govern reproductive development. RIN, a master regulator of fruit ripening, directly activates ethylene biosynthesis genes, including *ACS2* and *ACS4*, establishing a direct link between MADS-box activity and ethylene production [62,63]. RIN and TAGL1 has been shown to regulate genes involved in carotenoid and ABA metabolism, connecting carpel identity factors to stress hormone pathways [10,62,63]. Beyond these well-characterized examples, emerging evidence indicates that MADS-box genes broadly influence hormonal homeostasis: *SlMADS50* modulates IAA, GA3, and brassinosteroid contents, with corresponding changes in hormone biosynthesis and response gene expression [97]. These findings collectively demonstrate that MADS-box transcription factors function as upstream regulators of hormone metabolism and signaling throughout reproductive development.

Hormonal regulation exhibits pronounced spatiotemporal specificity. Multi-omics analyses reveal that precise hormone homeostasis, achieved through mechanisms such as conjugation, is essential for both development and stress adaptation. A clear example is found in stamens, where *GRETCHEN HAGEN* 3 (*GH3*) genes (*GH3-2*, *GH3-7*, and *GH3-15*) conjugate active auxin before anthesis to promote pollen thermotolerance. Disrupting this process in *gh3* mutants increases free auxin, alters the heat-stress proteome, and reduces pollen viability [98]. Conversely, during fruit ripening, the *SlGH3-2* modulates auxin–ethylene crosstalk. Its silencing elevates auxin, dysregulates ethylene and carotenoid pathways, and delays normal ripening while extending shelf life [99]. These findings collectively underscore that hormone conjugation acts as a critical integrator of developmental programs and environmental signals, ensuring reproductive success.

During fruit set and ripening initiation, intensive hormonal crosstalk further reinforces developmental robustness. The *BES1*/*BZR1* transcription factor *SlBES2* forms a bidirectional feedback loop with ABA, coordinating ABA accumulation and *SlBES2* activity to stabilize ripening progression [100]. Moreover, *BES1* acts as a transcriptional integrator linking brassinosteroid signaling with ethylene biosynthesis and core ripening regulators (*RIN*, *TAGL1*), thereby coupling developmental and metabolic networks [101].

Compared with Arabidopsis, tomato exhibits a more complex hormone-centered regulatory network in which auxin, GA, ethylene, and cytokinin influence inflorescence architecture, flower number, and fruit set [95,102,103]. For instance, auxin and GA act synergistically to promote fruit set and development, with ARF transcription factors such as *SlDOF9* and *SlARF9* serving as key integrators of these hormone signals [95]. This expanded hormonal–environmental integration likely reflects evolutionary optimization in fleshy fruit crops to balance developmental stability with environmental responsiveness.

In summary, tomato reproductive development is governed by multilayered coordination among: (1) spatiotemporal hormone metabolism; (2) inter-pathway feedback loops (e.g., SlBES2–ABA); (3) stage-dependent shifts in dominant hormonal regulators; and (4) dynamic integration of environmental inputs into hormone-centered transcriptional networks. Together, these mechanisms form a flexible regulatory architecture that ensures orderly progression from floral initiation to fruit maturation while enabling adaptive plasticity under stress conditions.

## 5. Conclusions and Future Prospects

Tomato reproductive development, from the vegetative shoot apical meristem through inflorescence commitment, floral organogenesis, and ultimately fruit formation, is orchestrated by a highly coordinated molecular regulatory network. The transition to the inflorescence meristem and the subsequent establishment of the floral meristem represent critical developmental transitions that determine inflorescence architecture, floral organ identity, and ultimately fruit yield and quality. As summarized in Table 1, significant progress has been made in identifying key transcription factors and hormonal regulators governing these processes.

Beyond fundamental developmental significance, tomato floral organ variations possess direct practical value for crop improvement. Several distinct floral phenotypes have been successfully exploited for breeding applications. Stigma exsertion facilitates outcrossing and eliminates manual emasculation, providing a valuable tool for hybrid seed production. Multi-carpel flowers, controlled by genes involved in meristem maintenance, correlate directly with increased fruit size and have been key targets in large-fruited tomato breeding programs. Cleistogamy, resulting from a recessed stigma, ensures efficient self-pollination in cultivated tomato. Male sterility mutants serve as essential components of hybrid seed production systems. These examples illustrate that targeted manipulation of floral organ development directly translates into agronomic gains, confirming that flower development is the starting point for fruit trait improvement.

Despite these advances, notable research gaps remain. The interactive mechanisms among genes, hormones, and environment in floral patterning are not fully understood. The regulatory network controlling male sterility exhibits functional redundancy requiring systematic dissection. Most importantly, the translation of specialized floral phenotypes into breeding applications remains insufficient, with many studies confined to descriptive observations rather than functional implementation.

The field is now poised for a conceptual shift: flower development can no longer be viewed as an endpoint but rather as the foundation for fruit traits. Genetic improvement of fruit characteristics should be redefined as delayed outputs of the floral regulatory network. Several unresolved questions define the frontier of tomato reproductive biology. How do floral meristem regulators interface with cell cycle machinery to coordinate carpel development and subsequent fruit formation? Does a floral–fruit transcriptional memory transmit carpel identity to the mature fruit? How are environmental signals integrated into floral developmental programs to influence reproductive success and yield stability?

Addressing these questions will require integrating multi-omics approaches with systematic functional validation. Single-cell transcriptome atlases tracing development from meristem to fruit can reveal regulatory dynamics connecting floral patterning to fruit outcomes. Identifying hub genes that link floral development to fruit traits through integrated analyses will uncover priority targets for breeding interventions. By bridging basic research to breeding practice through molecular marker-assisted selection or gene editing, fundamental discoveries will enable precise improvement of tomato yield and quality, fulfilling the promise of developmental biology for crop improvement.

## Figures and Tables

**Table 1:** List of some key genes involved in tomato flower and fruit development.

Gene	Function	Effect in Tomato	Reference
*TAGL1*	MADS-box family transcription factor gene	Silencing results in abnormal fruit development	[20]
*SISTER OF TM3*	MADS-box family transcription factor gene	Loss-of-function leads to abnormal inflorescence architecture	[12,30]
*SlWUS*	WUSCHEL (WUS) homeobox transcription factor family gene	Knockout results in reduced locule and carpel number	[36,37]
*SlMBP21*	MADS-box family transcription factor gene	Silencing induces sepal elongation	[45]
*SlCMB1*	MADS-box family transcription factor gene	Transgenic plants exhibit elongated peduncles	[46]
*SlMADS48*	MADS-box family transcription factor gene	Overexpression induces sepal elongation	[48]
*TAP3*	MADS-box family transcription factor gene	Loss-of-function leads to petal-to-sepal and stamen-to-carpel homeotic conversions	[49]
*SlAMS*	bHLH family transcription factor gene	Mutation causes pollen developmental defects	[55]
*TM6*	B-class MADS-box gene	loss-of-function causes abnormal stamens, exerted stigmas, and male sterility	[52]
*TPI (SlGLO2)*	B-class MADS-box gene	loss-of-function causes twisted stamens, exposed stigmas, and complete male sterility	[104]
*SlMS10*	bHLH family transcription factor gene	Mutation leads to tapetum dysfunction and microspore degeneration	[58]
*SlMSH2*	MutS family gene	Loss-of-function causes meiotic arrest/aberration and failure to produce normal pollen	[59]
*SlPIF3*	bHLH family transcription factor gene	Mutation results in prophase I arrest in pollen mother cells	[56]
*SlCIN2*	β-Fructofuranosidase family gene	Silencing impairs pollen development and maturation	[57]
*STIG1*	Subtilisin-like serine protease family gene	Silencing impairs pollen tube growth and seed set	[67]
*SlCRCa*, *SlCRCb*	YABBY family transcription factor gene	Functional redundancy; mutation causes indeterminate carpel growth and ‘fruit-in-fruit’ phenotype	[68]
*SlYABBY2a*	YABBY family transcription factor gene	Mutation induces septum invagination	[72]
*SlMYB3R3*	MYB family transcription factor gene	Mutation leads to abnormal fruit shape	[73]
*SlDOF10*	DOF transcription factor gene	Mutation causes abnormal ovary development	[81,93]
*SlARF7*	Auxin response factors (ARFs)	Downregulated expression leads to parthenocarpic fruit development	[105]
*SlNCED1*	Key rate-limiting enzyme in ABA	Overexpression and suppression of SlNCED1 lead to the abnormal development of anther/pollen	[106]
*SlSUS3*	Key enzymes for sucrose synthesis	Silencing *SlSUS3* decreased the number of flowers and fruits, and the proportion of multi-petal flowers	[107]

## Data Availability

No new data were created or analyzed in this study. Data sharing is not applicable to this article.
